# Demographic and clinical characteristics of people diagnosed with active sexually transmitted infections among monkeypox cases in Brazil: the 2022 outbreak

**DOI:** 10.1590/S1678-9946202466020

**Published:** 2024-04-05

**Authors:** Isabella Nepomuceno de Souza, Ana Roberta Pati Pascom, Matheus Funke Spinelli, Guilherme Borges Dias, Draurio Barreira, Angélica Espinosa Miranda

**Affiliations:** 1 Ministério da Saúde, Secretaria de Vigilância em Saúde e Ambiente, Departamento de HIV/Aids, Tuberculose, Hepatites Virais e Infecções Sexualmente Transmissíveis, Brasília, DF, Brazil; 2 Universidade Federal do Espírito Santo, Programa de Pós-Graduação em Doenças Infecciosas, Vitória, Espírito Santo, Brazil

**Keywords:** Sexually transmitted infections, Monkeypox, Surveillance

## Abstract

The worldwide monkeypox (mpox) outbreak in 2022 showed a high frequency of sexually transmitted infections (STI). A cross-sectional study was carried out using secondary data from the Brazilian official mpox surveillance systems. A total of 10,169 mpox cases were identified, with a median age of 32 years. Among them, 92.3% were male at birth and 57.5% were men who have sex with other men (MSM). Approximately 11% were diagnosed with STI, including 5.8% with syphilis and 2.5% with genital herpes. Individuals aged from 25 to 34 years, MSM, individuals with HIV-positive status, and those manifesting skin eruptions or penile edema were associated with STI. Laboratory investigation for mpox must be implemented as a priority in STI clinics (especially for MSM) to mitigate neglected cases, ensure appropriate treatments, and prevent misdiagnoses.

## INTRODUCTION

A high frequency of sexually transmitted infections (STI) has been reported in the 2022 global monkeypox (mpox) outbreak^1,2^, and sex or intimate contact was acknowledged as the critical transmission route^3^. Prior outbreaks of mpox have been associated with traveling to endemic areas in Western and Central Africa, zoonotic transfer by bodily fluid contact, and human-to-human via close contact with infectious lesions or bodily fluids, notably among household members and healthcare providers^3^.

Mpox classically shows a nonspecific systemic prodrome followed by a progressive vesicular rash. Differential diagnosis can be complicated by overlapping clinical symptoms with more common infectious agents but can ultimately be confirmed with laboratory analysis^4^. Mpox lesions can vary in number and distribution, though often concentrated on the face, oral mucosa, extremities, and genitals. In the current outbreak, painless anogenital lesions, generally appearing without a prodrome after sexual contact with infected persons, including men who have sex with men (MSM), have been reported^5^.

Although mpox human-to-human transmission can occur more frequently via respiratory droplets or direct contact with the mucocutaneous lesions of an infected individual^2,6^, classical mpox transmission requires close prolonged skin-to-skin contact with active lesions without the need for sexual contact^3,5^. Furthermore, mpox can also be considered an STI because an infected individual can spread the disease by body fluids and/or prolonged and intimate contact during sexual intercourse^3^.

This study aimed to compare demographic and clinical characteristics of mpox cases among people diagnosed with an active STI according to the mpox notification systems in Brazil to guide prevention strategies to stop the dissemination of the disease.

## MATERIALS AND METHODS

A cross-sectional study was conducted with nonidentified secondary data that were obtained from official notification surveillance systems (REDCap, e-SUS SINAN, e-SUS VS, and CeVeSP). REDCap and e-SUS SINAN are national systems, whereas e-SUS VS and CeVeSP are only used in their respective states (Espirito Santo and Sao Paulo)^7^. Probable and confirmed mpox cases in patients aged 15 years or above that were reported from June 07 to December 31, 2022, in Brazil, were included in this study. In short, confirmed cases referred to those with a positive or detectable mpox virus according to molecular diagnosis by real-time polymerase chain reaction (PCR) and/or sequencing. Probable cases included suspected cases with inconclusive test results or whose tests were not performed.

Social-demographic characteristics of confirmed and probable mpox cases were described as follows: region of residence (North, Northeast, Southeast, South, Central-West, missing, or declined to answer); sex assigned at birth (male, female, missing, or unavailable); gender (trans women, cis women, trans men, cis men, non-binary, other, missing, or unavailable); median age (1^st^ quartile - Q1 - and 3^rd^ quartile - Q3) and age group (15–24, 25–34, 35–49, 50+); self-reported ethnicity/skin color (White/Yellow, Brown/Black, Indigenous, and not informed). Those assigned as male at birth were stratified by sexual orientation (heterosexual, homosexual, bisexual, pansexual, other, not informed, assigned female at birth, missing, or declined to answer) and as gay men and men who have sex with men (MSM) (yes, no, assigned female at birth, missing, or declined to answer).

The STI prevalence rate among mpox notified cases was estimated as the proportion of confirmed and probable mpox cases diagnosed with syphilis, genital herpes, chlamydia, gonorrhea, or genital warts at the mpox notification. Clinical characteristics of mpox included: signs and symptoms (e.g., fever; rash; headache; adenomegaly; genital, oral or mucosal lesion; and penile edema); HIV/AIDS status at notification (yes, no, missing or unavailable); and immunosuppression, defined as the reduced capacity of the immune system to effectively respond to foreign antigens by any known pathological condition or use of pharmaceutical drug at the moment of mpox notification^7^ (yes, no, missing, or unavailable).

Descriptive statistics were used to assess the demographic and clinical characteristics of mpox cases diagnosed with at least one STI at the moment of mpox notification. Univariable and multivariable logistic regression models were applied to identify the factors associated with being diagnosed with an STI at mpox notification. Crude and adjusted odds ratios (OR and aOR) and 95% confidence intervals (95% CI) were estimated. All variables with a p-value<0.20 in the univariable analysis were included in the multivariable analysis. A final model was selected considering a p-value ≤.05 as the cutoff point for statistical significance.

This project was approved by the Research Ethics Committee at the Federal University of Espirito Santo, Nº 5.937.292/2023, in accordance with the National Health Council Resolution Nº 466 of December 12, 2012.

## RESULTS

As of December 2022, Brazil had 10,169 confirmed and probable cases of mpox, in individuals aged 15 and older (Table 1). The median age was 32 years (Q1=27 and Q3=38), 46.3% (n=4,706) were 25–34 years old and 34.5% (n=3,508), 35–49. A total of 92.3% (n=9,386) were assigned as male at birth, 72.1% (n=7,328) were cisgender males, and 57.5% (n=5,844) were classified as MSM. Most cases (61.5%, n=6,253) occurred in the Southeast, the wealthiest and most populated/populous geographical region in Brazil^8^. This study found no difference according to ethnicity/color, and 40.4% of the sample (n=4,113) were White/Yellow and 38.7% (3,935), Brown/Black.

**Table 1:** Demographic characteristics of confirmed and probable mpox cases, according to sexual transmitted infections diagnosis. Brazil, 2022.

Demographic characteristic	Mpox cases		Mpox cases diagnosed with an STI % cases with an STI	
	N	%	N	%
**Total**	10,169	100.0	1,099	10.8
**Sex at birth**				
Male	9,386	92.3	1,071	11.4
Female	762	7.5	28	3.7
NA	21	0.2	0	0.0
**Gender**				
Transgender women or tranvestites	54	0.5	10	18.5
Cisgender female	648	6.4	25	3.9
Cisgender male	7,328	72.1	904	12.3
Non-binary	78	0.8	13	16.7
Other	257	2.5	36	14.0
Missing/NA	1,804	17.7	111	6.2
**Age**				
Md (Q1-Q3)	32 (27–38)		32 (27–37)	
15-24	1,431	14.1	131	9.2
25-34	4,706	46.3	571	12.1
35-49	3,508	34.5	368	10.5
50+	524	5.2	29	5.5
**Ethnicity/skin color**				
White/Yellow	4,113	40.4	407	9.9
Black	3,935	38.7	521	13.2
Indigenous	16	0.2	2	12.5
Missing/NA	2,105	20.7	169	8.0
**Region of residence**				
North	482	4.7	84	17.4
Northeast	1,241	12.2	111	8.9
Southwest	6,253	61.5	611	9.8
South	1,044	10.3	131	12.5
Central-West	1,127	11.1	157	13.9
vMissing/NA	22	0.2	5	22.7
**Sexual orientation**				
Heterosexual	768	7.6	56	7.3
Homosexual	3,536	34.8	531	15.0
Bisexual	575	5.7	85	14.8
Pansexual	76	0.7	12	15.8
Other	410	4.0	29	7.1
Female at birth	762	7.5	28	3.7
Missing/NA	4,042	39.7	358	8.9
Men who have sex with men*				
No	1,007	9.9	67	6.7
Yes	5,844	57.5	792	13.6
Female at birth	783	7.7	28	3.6
Missing/NA	2,535	24.9	212	8.4

NA = Not available; Md = Median; Q1 = 1^st^ quartile; Q3 = 3^rd^ quartile; STI = Sexually transmitted infection; *Individuals assigned as male at birth who reported sex only with other men or with men and women were considered MSM.

At the moment of mpox notification, 10.8% (n=1,099) cases also showed at least one STI (Table 2). This study also observed a 5.8% (n=587) prevalence of syphilis, 2.5% (n=252) of genital herpes, and 0.4% of chlamydia (n=45), gonorrhea (n=43), and genital wart (n=41).

**Table 2:** Clinical characteristics of confirmed and probable mpox cases according to sexual transmitted infections diagnosis. Brazil, 2022.

Demographic characteristic	Mpox cases		Mpox cases diagnosed with an STI % cases with an STI	
	N	%	N	%
**Total**	10,169	100.0	1,099	10.8
**STI**				
Syphilis	587	5.8		
Genital herpes	252	2.5		
Chlamydia	45	0.44		
Gonorrhea	43	0.42		
Genital warts	41	0.40		
Other	131	1.3		
None	3,871	38.1		
NA	5,199	51.1		
**Immunosuppressed**				
No	5,104	50.2	476	9.3
Yes	2,869	28.2	549	19.1
Missing/NA	2,196	21.6	74	3.4
**Living with HIV**				
No	4,055	39.9	365	9.0
Yes	3,595	35.4	648	18.0
Missing/NA	2,519	24.8	86	3.4
**Hospitalization**				
No	4,133	40.6	511	12.4
Yes	114	1.1	17	14.9
Missing/NA	5,922	58.2	571	9.6
**Symptoms**				
At least one	9,449	92.9	1,059	11.2
Bleeding	120	1.2	24	20.0
Penile edema	489	4.8	88	18.0
Mucosa lesion	350	3.4	59	16.9
Genital legion	2,280	22.4	335	14.7
Oral lesion	550	5.4	80	14.5
Generalized lymphadenopathy	1,335	13.1	181	13.6
Sweating/shivering	1,411	13.9	184	13.0
Anal lesion	1,993	19.6	258	12.9
Rash	4,640	45.6	591	12.7
Sore throat	1,448	14.2	183	12.6
Adenomegaly	3,767	37.0	466	12.4
Fever	5,950	58.5	697	11.7
Other symptoms	6,729	66.2	787	11.7

STI = Sexually transmitted infection; NA = Not available

A more significant proportion of STI was diagnosed among cases assigned as male at birth (11.4%), transgender women (18.5%), and non-binary people (16.7%), individuals aged 25–34 years (12.1%), and those self-reported as Brown/Black (13.2%) and Indigenous (12.5%). STI prevalence was higher among homosexual (15.0%), bisexual (14.8%), and pansexual (15.6%) individuals, and those males at birth classified as MSM (13,6%) (Table 1).

Clinical aspects showed a greater STI prevalence among mpox cases, 19.1% were immunosuppressed and lived with HIV (18.0%). Moreover, STI prevalence totaled 20% among those with bleeding; 18%, for those with penile edema; and 16.9%, for those with mucosal lesions (Table 3).

**Table 3:** Results of univariable and multivariable logistic regression models for being diagnosed with a sexual transmitted infection at mpox notification. Brazil, 2022.

Demographic and clinical characteristic	OR(95%CI)	aOR(95%CI)
**Sex at birth**		
Male	3.38(2.30–4.95)	
Female	1.00	NS
NA	-	
**Gender**		
Transgender women or tranvestites	5.66(2.56–12.54)	2.24(0.85–5.91)
Cisgender female	1.00	1.0
Cisgender male	3.51(2.34–5.26)	1.45(0.72–2.92)
Non-binary	4.98(2.43–10.21)	1.93(0.77–4.80)
Other	4.06(2.38–6.92)	1.86(0.86–4.03)
Missing/NA	1.63(1.05–2.55)	1.02(0.50–2.06)
**Age**		
15-24	1.72(1.14–2.61)	1.85(1.21–2.84)
25-34	2.36(1.60–3.47)	2.02(1.36–3.00)
35-49	2.00(1.35–2.95)	1.70(1.14–2.53)
50+	1.00	1.0
**Ethnicity/skin color**		
White/Asian	1.00	1.0
Black	1.39(1.21–1.59)	1.26(1.08–1.46)
Indigenous	1.30(0.29–5.74)	1.13(0.25–5.19)
Missing/NA	0.79(0.66–0.96)	0.92(0.76–1.13)
**Region of residence**		
North	2.15(1.58–2.92)	1.55(1.13–2.12)
Northeast	1.00	1.00
Southwest	1.10(0.89–1.37)	1.37(1.08–1.73)
South	1.46(1.12–1.91)	1.48(1.11–1.95)
Central-West	1.65(1.27–2.13)	1.37(1.05–1.79)
Missing/NA	2.99(1.08–8.27)	4.54(1.5–13.78)
**Sexual orientation**		
Heterosexual	1,0	NS
Homosexual	2.25(1.69–2.99)	
Bisexual	2.21(1.54–3.15)	
Pansexual	2.38(1.22–4.68)	
Other	0.97(0.61–1.54)	
Female at birth	0.49(0.3–0.77)	
Missing/NA	1.24(0.92–1.66)	
Men who have sex with men*		
No	1.00	1.00
Yes	2.20(1.70–2.85)	1.57(1.20–2.05)
Female at birth	0.52(0.33–0.82)	0.84(0.42–1.70)
Missing/NA	1.28(0.97–1.70)	1.16(0.86–1.57)
**Immunosuppressed**	1.0	
No	2.30(2.02–2.75)	NS
Yes	0.34(0.26–0.44)	
Missing/NA	1.0	
**Living with HIV**	1.0	1.0
No	2.22(1.94–2.55)	1.72(1.36–2.18)
Yes	0.36(0.28–0.45)	0.64(0.48–0.85)
Missing/NA	1.0	1.0
**Hospitalization**		
No	1.0	1.0
Yes	1.58(1.23–2.02)	1.51(1.16–1.95)
Missing/NA	0.73(0.60–0.88)	0.89(0.72–1.09)
Symptoms**		
Bleeding	2.09(1.33–3.28)	NS
Penile edema	1.88(1.48–2.39)	1.34(1.04–1.74)
Mucosa lesion	1.71(1.28–2.28)	NS
Genital legion	1.61(1.40–1.84)	NS
Oral lesion	1.44(1.12–1.84)	NS
Generalized lymphadenopathy	1.35(1.14–1.60)	NS
Sweating/shivering	1.29(1.09–1.52)	NS
Anal lesion	1.30(1.12–1.51)	NS
Rash	1.44(1.27–1.64)	1.22(1.04–1.42)
Sore throat	1.23(1.04–1.46)	NS
Adenomegaly	1.29(1.13–1.46)	NS
Fever	1.26(1.11–1.43)	NS
Other symptoms	1.33(1.16–1.52)	NS

OR = Odds Ratio; aOR = Adjusted Odds Ratio; Md = Median; Q1 = 1^st^ quartile; Q3 = 3^rd^ quartile; STI = Sexually transmitted infection; NA = Not available; NS = Not-significant; *Individuals assigned as male at birth who reported sex only with other men or with men and women were considered MSM; **The reference category for the aOR and OR estimation for all symptoms were “did not present this symptom”.

In the adjusted analyses (Table 1), MSM (aOR=1.57; 95%CI: 1.20–2.05), age groups 15–24-year olds (aOR=1.85; 95%CI: 1.21–2.84) and 25–34-year olds (OR=2.02; 95%CI: 1.36–3.00), Brown/Black individuals (aOR=1.26; 95%CI: 1.08–1.46), and those living in the North (aOR=1.55; 95%CI: 1.13–2.12) or the South (aOR=1.48; 95%CI: 1.11–1.95) were more likely to being diagnosed with an STI. Patients living with HIV (aOR=1.72; 95%CI: 1.36–2.18), who were hospitalized (aOR=1.51; 95%CI: 1.16–1.95), and who showed either skin eruption (aOR=1.22; 95%CI: 1.04–1.42) or penile edema (aOR=1.34; 95%CI: 1.04–1.74) were also associated with having an STI.

## DISCUSSION

Our results showed a high frequency of STI among cases of mpox in Brazil. Syphilis and genital herpes, followed by chlamydia, gonorrhea, and genital warts were the most frequent STI. These results corroborate other studies, which also described high rates of STI among mpox confirmed cases^1,6,9^.

Even though the most affected population is known and is the same in all countries in which mpox is not endemic, the behavior of the current outbreak, mainly transmitted during sexual contact, is a concern. Our study showed that, in Brazil, the odds of having an STI at mpox notification is higher among MSM, and Black/Brown people, an already vulnerable population with worst health indicators^10,11^.

Furthermore, this study showed that the odds of having an STI were higher among those mpox cases with HIV. Other studies showed that severe mpox and associated complications were higher in the presence of HIV, especially in cases with AIDS^1,8,12^. Concomitant sexual transmitted infections, including HIV, should be investigated in all suspected mpox patients and classical sexually transmitted diseases should also be considered. Differential STI diagnosis is essential due to the distinct clinical management of patient, treatment, and control of outbreaks of different STI^1,3,6^.

Mpox symptoms may simulate other common infectious etiologies, including herpes simplex, varicella-zoster, syphilis, lymphogranuloma venereum, chancroid, gonorrhea, chlamydia, and smallbow^13^. In this study, of all analyzed mpox symptoms, skin eruption or penile edema were positively associated with having an STI. Even though some of the symptoms described in the study showed no statistical differences, systemic symptoms (adenomegaly, fever, and headache) are common in other STI, making it essential to align strategies for prevention and health promotion to reduce misdiagnoses and the likelihood of the co-infection of mpox and STI^14^.

This study holds some limitations, mainly related to the fact that it is a study with an analysis of Brazilian national surveillance data due to its intrinsic possibility of information bias. However, the completeness of the data regarding some variables may configurate a problem. The Brazilian Ministry of Health has well-established parameters for notification and testing that are described in national protocols to enforce a successful approach to care for mpox cases in any healthcare facility allied to a laboratorial surveillance strategy^15^. It is noteworthy that a large sample was used in this study, with standardized and unique forms for all services that care for and notify mpox cases, making it possible to analyze the national data in a field that remains scarcely studied.

## CONCLUSIONS

Our study used national surveillance data to show the association between mpox and other STI. Maintaining and improving regular surveillance is a key component of controlling mpox and preventing new outbreaks. Moreover, some mpox symptoms may overlap with those of other STI. Therefore, healthcare providers must be aware of clinical signs to mitigate the occurrence of neglected cases, ensure appropriate treatments, and prevent erroneous diagnoses. Laboratory screening for HIV, syphilis, hepatitis, and other STI as an offer of combined prevention measures, should be considered for persons evaluated for mpox, especially among MSM.
